# A droplet digital PCR assay for detection and quantification of *Verticillium nonalfalfae* and *V. albo-atrum*

**DOI:** 10.3389/fcimb.2022.1110684

**Published:** 2023-01-11

**Authors:** Di Wang, Enliang Liu, Haiyang Liu, Xi Jin, Chunyan Niu, Yunhua Gao, Xiaofeng Su

**Affiliations:** ^1^ Center for Advanced Measurement Science, National Institute of Metrology, Beijing, China; ^2^ Institute of Grain Crops, Xinjiang Academy of Agricultural Sciences, Urumqi, China; ^3^ Institute of Plant Protection, Xinjiang Academy of Agricultural Sciences, Urumqi, China; ^4^ Hebei Technology Innovation Center for Green Management of Soil-Borne Diseases, Baoding University, Hebei, China; ^5^ Biotechnology Research Institute, Chinese Academy of Agricultural Sciences, Beijing, China

**Keywords:** droplet digital PCR, quantitative real-time PCR, diagnostic performance, accuracy, precision

## Abstract

*Verticillium nonalfalfae* and *V. albo-atrum* are notorious pathogenic fungi that cause a destructive vascular disease called Verticillium wilt worldwide. Thus, timely and quantitative monitoring of fungal progression is highly desirable for early diagnosis and risk assessment. In this study, we developed a droplet digital polymerase chain reaction (ddPCR) assay to detect and quantify *V. nonalfalfae* and *V. albo-atrum*. The performance of this assay was validated in comparison with that of a quantitative real-time polymerase chain reaction (qPCR) assay. The standard curve analysis of the ddPCR assay showed good linearity. The ddPCR assay indicated similar detection sensitivity to that of qPCR on pure genomic DNA, while it enhanced the positive rate for low-abundance fungi, especially in alfalfa stems. Receiver operating characteristic analysis revealed that ddPCR provided superior diagnostic performance on field tissues compared to qPCR, and the area under curve values were 0.94 and 0.90 for alfalfa roots and stems, respectively. Additionally, the quantitative results of the two methods were highly concordant (roots: R^2^ = 0.91; stems: R^2^ = 0.76); however, the concentrations determined by ddPCR were generally higher than those determined by qPCR. This discrepancy was potentially caused by differing amplification efficiencies for qPCR between cultured and field samples. Furthermore, the ddPCR assays appreciably improved quantitative precision, as reflected by lower coefficients of variation. Overall, the ddPCR method enables sensitive detection and accurate quantification of *V. nonalfalfae* and *V. albo-atrum*, providing a valuable tool for evaluating disease progression and enacting effective disease control.

## 1 Introduction

Verticillium wilt (VW), a major vascular plant disease, causes considerable yield and economic losses worldwide. Typical symptoms of VW are plant stunting, discoloration, wilting, and eventual mortality (Klosterman et al., 2011). VW is caused by soil-borne and pathogenic *Verticillium* spp., mainly *V. nonalfalfae* and *V. albo-atrum*, which both have a broad host range (Pegg and Brady, 2002). *V. nonalfalfae* (a relatively new species in the genus) can infect potato, spinach, solanaceous crops, and trees (Flajsman et al., 2017). The primary hosts of *V. albo-atrum* are cotton, potato, tomato, hop, ornamental plants, fruit trees, and, especially, alfalfa. Among of them, alfalfa, an important forage resource with high yield and good palatability, is widely planted around the world and known as “the king of forage”. In the United States and Canada, VW is among the most destructive diseases in alfalfa (Larsen et al., 2007). The quarantine of all the alfalfa products from abroad was forcible in China (Announcement No. 351 of the Ministry of Agriculture and Rural Affairs of PRC, 2020). In the field, dormant structures (microsclerotia) are triggered to germinate by exudates from the roots of the host plant. Subsequently, hyphae infect the host through the root hairs or lateral roots (Fradin and Thomma, 2006). After invading the xylem vessels, the hyphae infest the adjacent vasculature and grow along the vascular system toward the tip of the plant (Zhao et al., 2014; Su et al., 2018). Ultimately, the pathogen reproduces and blocks the vascular system, interfering with the transport of water throughout the host and causing wilt symptoms (Floerl et al., 2008; Wang et al., 2021).

Given the tremendously devastating effects and the difficulty of prevention, developing an accurate method to qualitatively and quantitively detect these two fungi provides an effective and economical management strategy. Traditional methods of isolation and biological assays have long operation cycles, poor time efficiency, and relatively low sensitivity, and immunological methods exhibit cross-reactivity, poor reproducibility, and cumbersome operation steps (Isaac, 1946; Skadow, 1966; Griffiths, 1971). Recently, quantitative real-time polymerase chain reaction (qPCR) assays have been widely implemented for pathogen load testing, with greatly improved sensitivity and shortened detection times (Pasche et al., 2013). However, quantification by qPCR depends on external standard curves, which are a major source of variability (Bustin et al., 2009). In practice, applicable reference standards are often not readily available except for the most common target analytes; furthermore, qPCR efficiency often varies within and between laboratories (Svec et al., 2015). Thus, a lack of standardization and relatively poor measurement precision limits its usefulness.

Digital polymerase chain reaction (dPCR) is an emerging technique that performs absolute quantification by counting nucleic acid molecules, providing a simple, standardized quantification strategy (Huggett et al., 2015). It has potentially unique advantages over qPCR, including absolute quantification in a “calibration-free” manner and robustness to variations in PCR efficiency (Sanders et al., 2011). Furthermore, sample partitioning enables dPCR to tolerate inhibitors and decreases background noise, thereby reducing false negatives (Alikian et al., 2017). Additionally, perhaps most importantly, dPCR is reported to improve accuracy and precision in quantification in comparison with qPCR (Pinheiro et al., 2012; Hindson et al., 2013). Therefore, digital PCR technique holds promise as a highly sensitive and accurate measurement method (Baker, 2012).

Although dPCR is increasingly utilized to quantify target nucleic acids in various fields (Whale et al., 2012; Félix-Urquídez et al., 2016; Salipante and Jerome, 2020), its application for the quantitative detection of plant pathogens has been limited; moreover, the systematic evaluation of its performance against a background matrix is understudied. In this study, we established and validated a sensitive and accurate droplet digital PCR (ddPCR) system for the detection and quantification of *V. nonalfalfae* and *V. albo-atrum*, to explore whether this method has the advantages over the analog qPCR assay for plant pathogen diagnosis. Key performance parameters, including diagnostic performance, detection sensitivity, quantitative agreement, accuracy, and repeatability regarding field samples, for the ddPCR assay were assessed in comparison with standard qPCR.

## 2 Materials and methods

### 2.1 Fungal, bacterial, soil, and plant samples

The *V. nonalfalfae* and *V. albo-atrum* strains were stored in our laboratory. Eight fungal isolates (*Exserohilum turcicum*, *V. nigrescens*, *Magnaporthe oryzae*, *Bipolaris maydis*, *Rhizoctonia cerealis*, *Fusarium oxysporum* f. sp. *conglutinans*, *Ustilaginoidea virens*, and *Fusarium pseudograminearum*) and five bacterial isolates (*Acidovorax citrulli*, *Xanthomonas oryzae* pv. *oryzae*, *Pseudomonas syringae*, *Ralstonia solanacearum*, and *Xanthomonas campestris* pv. *campestris*) were obtained from the Chinese Academy of Agricultural Sciences and China Agricultural University. All samples were stored in 25% glycerol at -80°C.

Samples of alfalfa stems and roots were collected in an experimental field (Langfang, Hebei Province, China, 39°51’N, 116°60’E) and quickly frozen in liquid nitrogen. The plot had been sampled and tested numerous times previously for infection by *V. nonalfalfae* and *V. albo-atrum*; therefore, the infection status of each alfalfa plant was known.

We excised 200 mg fresh alfalfa stems and roots from each sample, respectively. They were each homogenized separately in 4 mL lysis buffer. Nucleic acid quality and quantity were determined using a NanoDrop 2000 spectrophotometer (Thermo Fisher Scientific, USA).

### 2.2 Phylogenetic tree construction

The internal transcribed spacer regions (ITS) sequences of *V. albo-atrum* (MH856937.1), *V. nonalfalfae* (KT362917.1), *V. alfalfae* (NR 126129.1), *V. longisporum* (MH864843.1), *V. nubilum* (MH856939.1), *Ilyonectria radicicola* (KM503139.1), *V. dahliae* (MH864842.1), *Volutella ciliata* (JQ647447.1), *V. zaregamsianum* (JN188008.1), *V. tricorpus* (MH857388.1), *V. klebahnii* (NR 126128.1), *V. tricorpus* (GO336726.1), *Acremonium nepalense* (LR590100.1), *Plectosphaerella cucumerina* (KM357306.1), and *Brunneochlamydosporium nepalense* (MH860634.1) were downloaded from the National Center for Biological Information (https://www.ncbi.nlm.nih.gov/). To construct a phylogenetic tree, the ITS sequences were compared using the ClustalW program (http://www.clustal.org/clustal2/), and the neighbor-joining approach was applied using the MEGA software (version 7.0.26, Auckland, New Zealand).

### 2.3 DNA extraction

The biological samples were re-cultured in complete medium and Luria-Bertani liquid medium at temperatures of 25°C and 37°C, respectively. Samples were harvested by centrifugation at 3000 ×g for 10 min. Subsequently, DNA was extracted following the manufacturer’s instructions (KG203; Tiangen, Beijing, China). DNA was extracted from alfalfa stems following the instructions of the Hi-DNAsecure Plant Kit (DP350; Tiangen, Beijing, China). Genomic DNA was isolated from the soil samples using a TIANamp Soil DNA Kit according to the manufacturer’s protocol (DP336; Tiangen, Beijing, China). The quality and concentration of genomic DNA were detected *via* 1% agarose gel electrophoresis and a NanoDrop 2000 spectrophotometer (Thermo Fisher Scientific, USA). Subsequently, samples were aliquoted and stored in refrigerator at -80°C.

The ITS sequences were amplified with the primers 5’-TCCGTAGGTGAACCTGCGG-3’ and 5’-TCCTCCGCTTATTGATATGC-3’ in *V. nonalfalfae* and *V. albo-atrum* strains and sequenced by Sangon Biotech Co., Ltd. (Shanghai, China). The results were aligned using the Basic Local Alignment Search Tool (https://blast.ncbi.nlm.nih.gov/Blast.cgi).

### 2.4 Primer and probe assessment

The primer/probe sets used in the qPCR and ddPCR experiments (**Table 1**) were preliminarily screened and evaluated to confirm the complete identity using only the target sequences.

**Table 1:** Primers and probes used in this study.

Name	Target	Oligo sequence (5’-3’)	Product length (bp)	Citation
va1	SCAR (DQ333342)	F: GGCCACGCTAGCCTTCACTA	75	(Larsen et al., 2007)
		R: CGAGTTCGCGGCAGGTA		
		P: TTTCGACCTGCCGTGCGCG		
va2	V357I (DQ266246.1)	F: GGCTTTTGCTTTCTCTTG	150	(Maurer et al., 2013)
		R: GACCAAATGTAATTGTCCAG		
		P: AGGTATAAGGTCCATATCCAACACGAG		
va3	tef1a (LR026334.1)	F: CGTACGATTGAGAAGTTTGAGATAAGTG	100	(General Administration of Customs of the People's Republic of China., 2019)
		R: CGTCGGAAACCATGAAAACA		
		P: CTGCTTGAATCTACAC		
va4	ITS (MH856937.1)	F: CCGGTACATCAGTCTCTTTA	330	(General Administration of Quality Supervision, Inspection and Quarantine of the People’s Republic of China., 2014)
		R: CTCCGATGCGAGCTGTAAT		
		P: ATGCCTGTCCGAGCGTCGTTTCA		

Probes were synthesized and labeled with 6-carboxy-fluorescein reporter dye and black hole quencher 1 on the 5’- and 3’-terminal nucleotides, respectively (Sangon Biotech). The concentration was diluted to 100 μM as stock solution, stored at -20°C, and diluted to 10 μM for the assay.

### 2.5 qPCR

The qPCR amplification was accomplished using the ABI 7500 Fast Real-Time PCR system (Applied Biosystems, Foster City, CA, USA). The final reaction was in a 20 μL volume containing 10 μL of 2×AceQ Universal U+ Probe Master Mix V2 (Vazyme Biotech Co., Ltd, Nanjing, China), 0.4 μL each of the forward and reverse primers, 0.2 μL of the probe solution, 2 μL of genomic DNA, and 7 μL of nuclease-free H_2_O. The amplification was performed as follows: denaturation at 95°C for 5 min, followed by 40 cycles of denaturation at 95°C for 10 s and annealing and elongation at 60°C for 50 s. The cycle threshold (Ct) values were calculated using 7500 software v2.3 (Applied Biosystems, USA).

A standard curve for qPCR was constructed using the genomic DNA of *V. nonalfalfae* and *V. albo-atrum*. DNA concentration was measured using a NanoDrop 2000 (Thermo Scientific). The standard curve was plotted according to the Ct values from qPCR for various sample amounts, which were obtained by serial dilution of the genomic DNA of *V. nonalfalfae* and *V. albo-atrum*.

### 2.6 ddPCR reaction

The ddPCR assay was optimized with respect to the annealing temperature, primer concentration, and probe concentration. Temperatures of 54°C, 56°C, 58°C, 60°C, and 62°C were examined using a Veriti™ 96-Well Thermal Cycler (Applied Biosystems, USA) with a primer/probe concentration of 500 nM/250 nM. Primer/probe concentrations of 600 nM/400 nM, 500 nM/250 nM, and 400 nM/100 nM were examined using a thermal cycler at 56°C.

The ddPCR reactions were performed using 500 nM solutions of each forward and reverse primer, a 250 nM solution of the 2 × ddPCR supermix for probes (Bio-Rad, Pleasanton, CA, USA) (after optimization), and 2 μL of genomic DNA. The total reaction volume was 20 μL. The reaction mixture was added to an 8-well cartridge using a droplet generator (Bio-Rad), and the wells were injected with 70 μL of droplet generation oil (Bio-Rad). The emulsion (70 μL) was transferred to a 96-well ddPCR plate (Bio-Rad) and amplified as follows with a ramp rate of 2°C s^−1^: denaturation at 94°C for 10 min, followed by 45 cycles of denaturation at 94°C for 30 s and annealing at 56 °C (optimized temperature) for 50 s, followed by a final denaturation at 98°C for 10 min. Subsequently, the 96-well plate was shifted to an QX200 droplet reader (Bio-Rad), and the data analysis was performed using QuantaSoft Version 1.7.4.0917 (Bio-Rad). The ddPCR analysis was performed in triplicate to accurately quantify the amounts of genomic DNA.

### 2.7 Linear regression curve of the ddPCR assay

Linear regression curves were constructed using ten-fold serial dilutions of genomic DNA as templates, ranging from 4 to 4 ×10^4^ copies/reaction and 6 to 6 ×10^4^ copies/reaction for *V. nonalfalfae* and *V. albo-atrum*, respectively. Three independent experiments were performed with four reactions at each concentration. The linear regression curves were generated by plotting the copy number concentrations measured by ddPCR against the expected dilution values.

### 2.8 Limit of quantification and limit of detection at 95% probability

The limit of quantification (LoQ) was determined as the lowest concentration where all runs were positive and the coefficient of variation (CV) of the measured copy number was up to 25% (Kralik and Ricchi, 2017).

The limit of detection at 95% probability (LoD95%) was defined as the lowest concentration at which at least 95% of the replicates produced positive results, regardless of the accuracy or precision (Forootan et al., 2017). The LoD95% of each assay was estimated using a probit analysis with the target concentration (dilution level) as an explanatory variable and the positive detection rate (positive/total) as a response variable (Corman et al., 2020). The probit analysis was conducted with 95% confidence intervals (95% CI) using the SPSS software (version 20.0; SPSS, Chicago, IL, USA).

To obtain the LoQ and LoD95% of the ddPCR and qPCR assays, a series of two-fold dilutions of genomic DNA (from 1.1 to 294 copies/reaction for *V. nonalfalfae* and from 1.8 to 236 copies/reaction for *V. albo-atrum*) were tested to ascertain the end-point dilutions. Two independent experiments were performed over two consecutive days, with five replicates of each concentration on each day (Pavšič et al., 2016). These experiments were tested in parallel, and the template DNA was only frozen and thaw once. The distilled water was used as a negative control.

### 2.9 Analytical specificity evaluation

A specificity evaluation panel was used to explore the specificity of the ddPCR assays for *V. nonalfalfae* and *V. albo-atrum* and included the eight fungal and five bacterial isolates listed in the “Fungal, bacterial, soil, and plant samples” section above.

### 2.10 Evaluation of diagnostic performance, quantitative agreement, and precision with filed samples

Receiver operating characteristic (ROC) curves were generated to compare the diagnostic performance of the ddPCR and qPCR assays. True positive alfalfa roots and stems that were infected with *V. nonalfalfae* or *V. albo-atrum* were used to generate the ROC curves based on previous data. ROC curves were constructed using the SPSS software (version 20.0; SPSS, Chicago, IL, USA).

Quantitative agreement between ddPCR and qPCR was investigated using the method of Bland and Altman (Bland and Altman, 1986), which utilized the log_10_-transformed concentration of each sample. The inhibition effect of the residual matrix on both ddPCR and qPCR assays was evaluated by quantifying a constant amount of gDNA in the presence of alfalfa stem and root extracts. We spiked the reactions with the same amount of gDNA from cultured isolates (*V. nonalfalfae*: 1.6 ×10^4^ ITS molecules; *V. albo-atrum*: 2.4×10^4^ ITS molecules), and 5 μl of extracts from healthy cotton stems and roots. The exact preparation process described by Maheshwari et al. (Maheshwari et al., 2017).

For each alfalfa field sample, CV values for ddPCR and qPCR were determined by calculating the standard deviation within each set of triplicates (n = 4) and dividing each of these by their respective average concentration values. The CV values for qPCR were determined using linear-scale copy number concentrations rather than the Ct values, which were directly comparable to those of ddPCR.

## 3 Results

### 3.1 Development of the ddPCR assay

**Figure 1:**
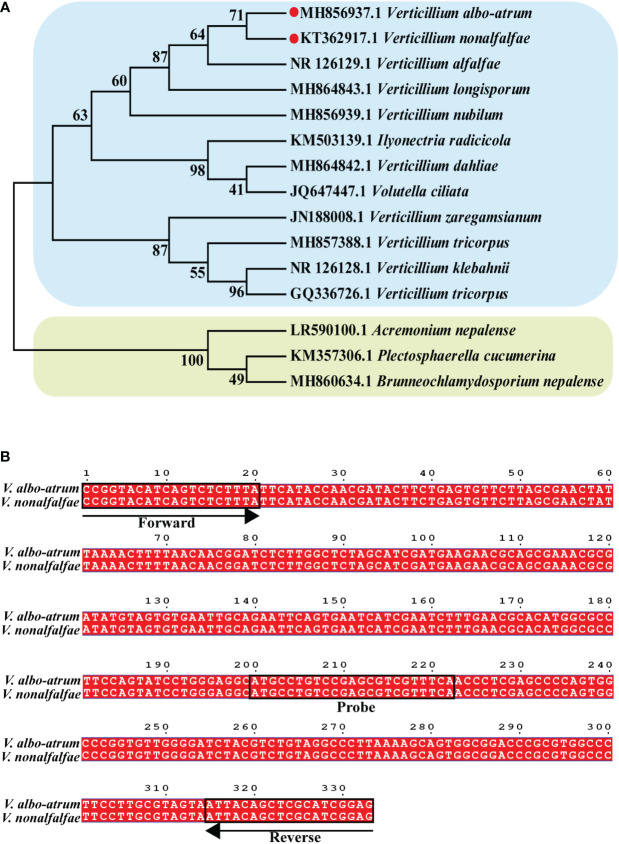
Construction of the phylogenetic tree for *Verticillium* and other fungi. **(A)** Binding sites of va4 primer/probe sets for ITS sequence in *V. nonalfalfae* and *V. albo-atrum* genomes **(B)**. Phylogenetic tree was constructed based on ITS sequence of 15 species of fungi, and the numbers at the nodes exhibit confidence level.

To analyze the position of *Verticillium* in a broader fungal phylogenetic context, the phylogenetic relationships among major *Verticillium* spp. and other fungi were analyzed using ITS sequences. Phylogenetic analysis indicated that the 15 selected species were divided into two distinct clades, and *V. nonalfalfae* and *V. albo-atrum* had the closest phylogenetic relationship (**Figure 1A**). This is consistent with the fact that both *V. nonalfalfae* and *V. albo-atrum* belong to *Verticillium* and share a similar pathogenic mechanism that results in symptoms of plant stunting, wilting, and early senescence. Hence, we aimed to develop a ddPCR assay that enables the quantitative detection of *V. nonalfalfae* and *V. albo-atrum* simultaneously, to improve the efficiency of fungal diagnoses.

To develop an optimal ddPCR assay, four reported primer/probe sets targeting the different regions of the two fungi (KT362917.1 and MH856937.1 in the Genebank database) were initially validated using qPCR systems. The results showed that Va4 primer/probe set performed better than the other primer/probe sets, obtaining the target amplicons with low Ct values for both fungal samples (**Table S1**; **Figure S1**). Based on the sequence alignment, the amplification sequence of the Va4 primer/probe set was identical for *V. nonalfalfae* and *V. albo-atrum* (**Figure 1B**). Therefore, Va4 primer/probe set was selected for further establishment of the ddPCR assay.

**Figure 2:**
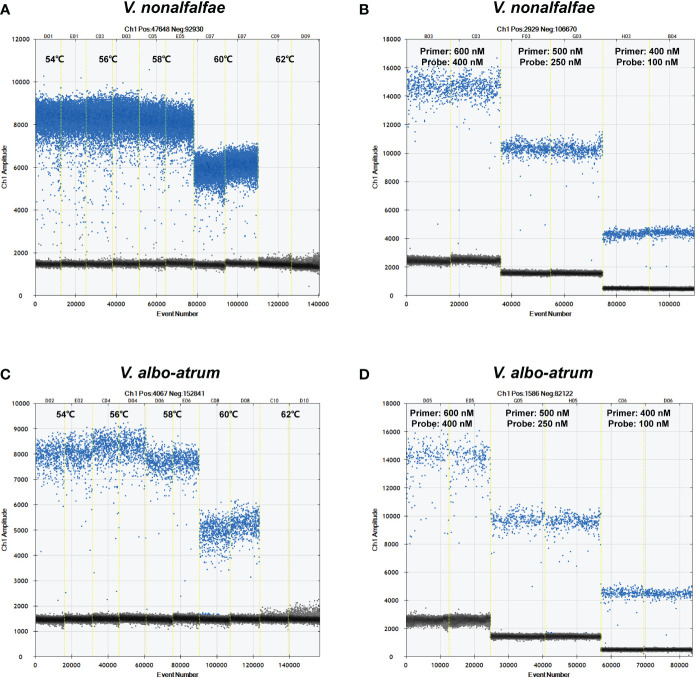
Optimization of annealing temperatures and primer/probe concentrations for *V. nonalfalfae* **(A, B)** and *V. albo-atrum.* **(C, D)** The blue spots represent positive droplets, and the gray spots represent negative droplets.

The ddPCR reaction conditions were optimized with respect to annealing temperature and primer/probe concentrations by evaluating five temperatures (ranging from 54°C to 62°C) and three primer/probe concentrations (600/400 nM, 500/250 nM, and 400/100 nM). As shown in **Figure 2**, the optimal annealing temperature and primer/probe concentrations were 56°C and 500/250 nM, respectively, which enabled the best separation of the positive and negative droplets, with a low abundance of “rain” (droplets falling between the positive and negative populations).

### 3.2 Dynamic range and specificity testing for the ddPCR assay

**Figure 3:**
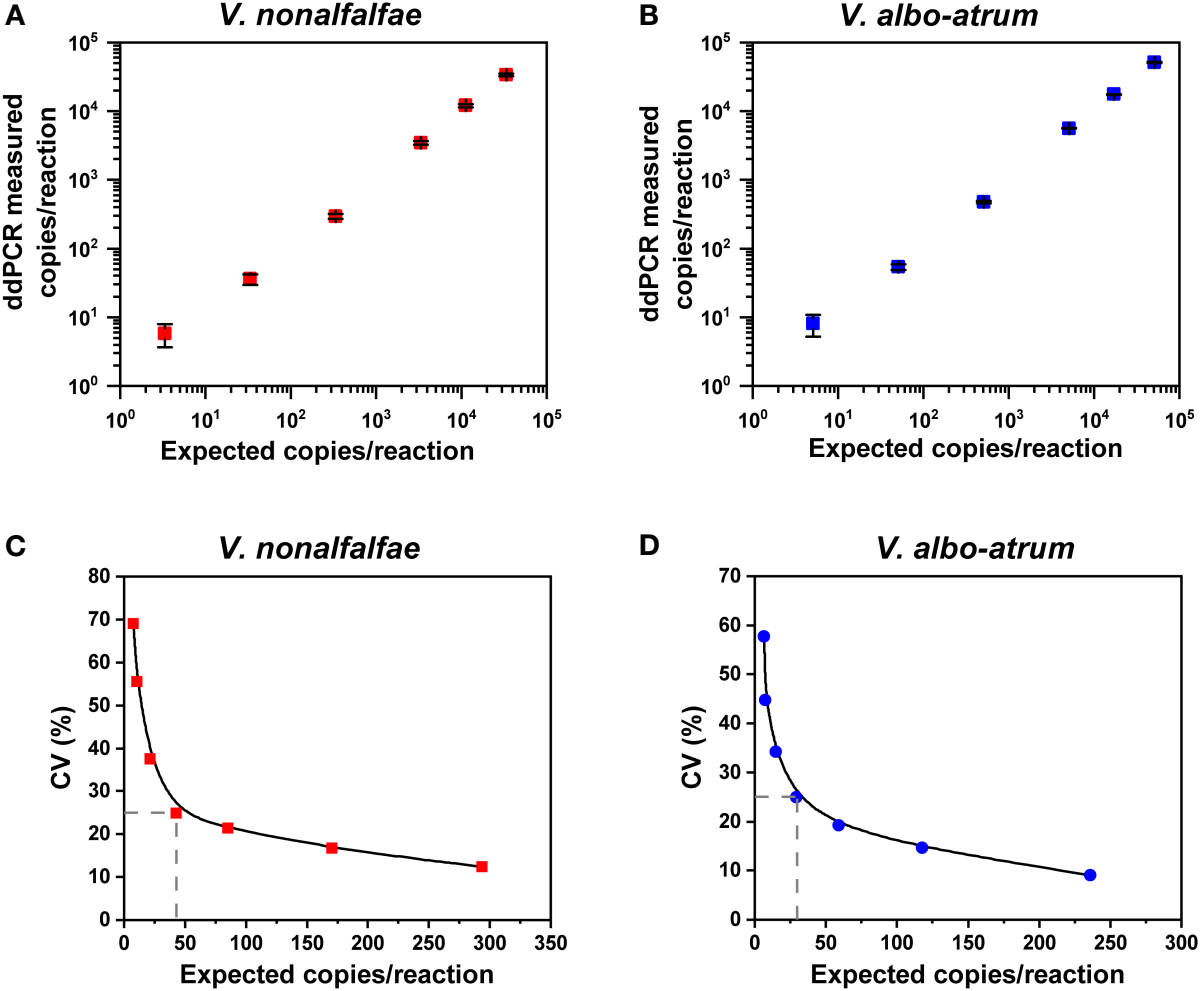
Linear range and precision analysis for ddPCR quantification of *V. nonalfalfae* and *V. albo-atrum*. **(A, B)** Linear regression of the ddPCR assays for *V. nonalfalfae* **(A)** and *V. albo-atrum* **(B)** (*p* < 0.0001). Data are shown as mean and standard deviation for each dilution series (n =4). **(C, D)** Inter-assay precision profiles for ddPCR at each low-concentration dilution for *V. nonalfalfae* **(C)** and *V. albo-atrum* **(D)** The L_O_Q was set at the copies/reaction that corresponded to a CV of 25% (intersecting dotted lines on the plots).

For the linear regression analysis, serial dilutions of genomic DNA extracted from the two cultured isolates were prepared. The ddPCR assays exhibited good linearity between the measured and anticipated values for each interval for *V. nonalfalfae* (R^2^ = 0.9996 and 0.94<Slope<1.03) and *V. albo-atrum* (R^2^ = 0.9997 and 0.96<Slope<1.04) over a dynamic range from approximately 10^1^ to 10^4^ copies/reaction (**Figures 3A, B**). The LoQ of the ddPCR assay was 43 and 30 copies for *V. nonalfalfae* and *V. albo-atrum*, respectively, which met the criterion for a LoQ with a CV lower than 25% (**Figures 3C, D**).

Specificity evaluation indicated that the runs were specific for *V. nonalfalfae* and *V. albo-atrum*, and no cross-reactivity was observed with the other eight similar fungal and five bacterial samples (**Table S2**).

### 3.3 Comparison of the LoD95% between ddPCR and qPCR on pure genomic DNA

**Figure 4:**
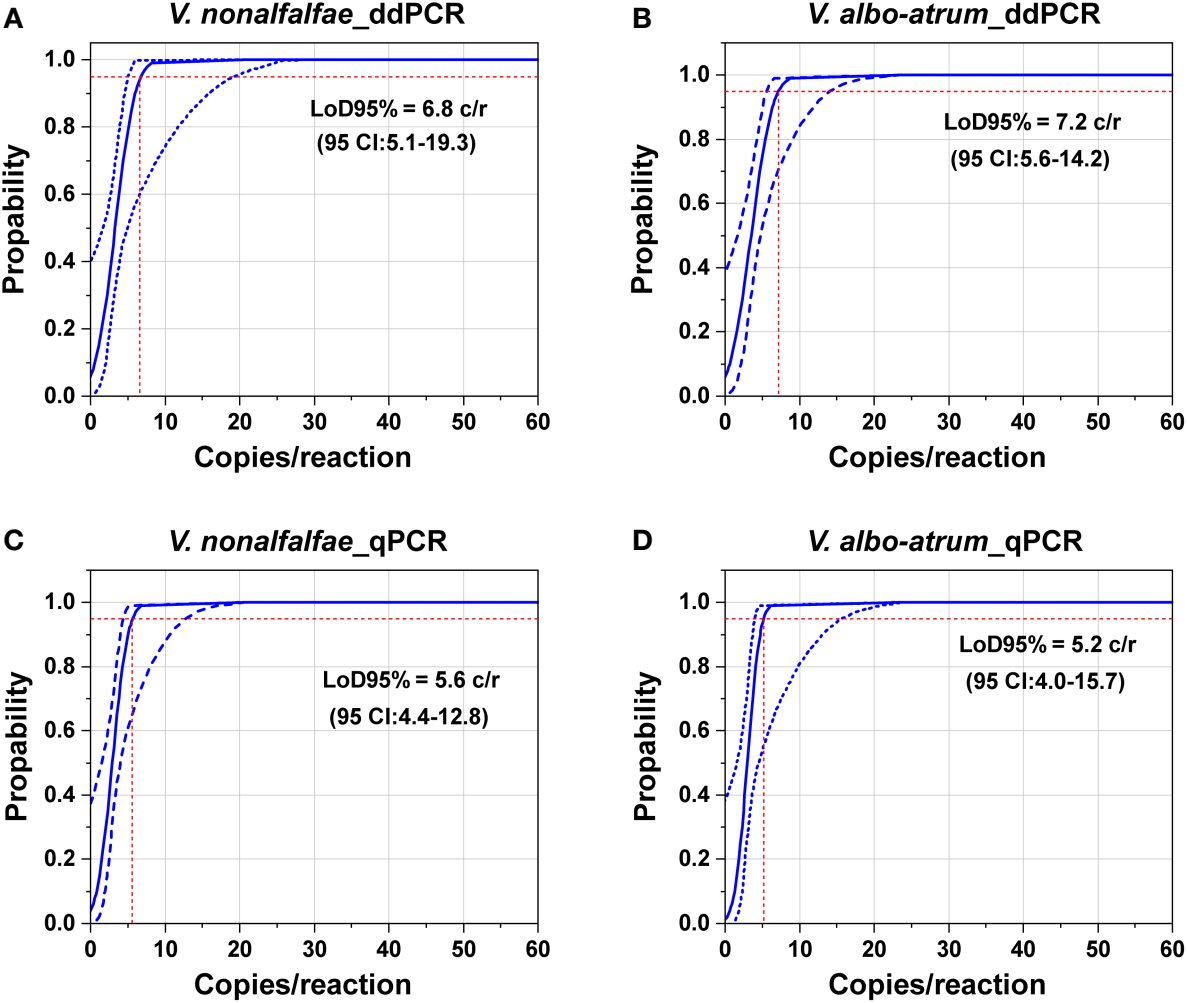
Comparation of the limits of detection of the ddPCR and qPCR assays based on pure genomic DNA of *V. nonalfalfae* and *V. albo-atrum*. Probit analysis determined the LoD95% of ddPCR **(A, B)** and qPCR **(C, D)**. The *y* axis shows the fraction of positive results in 10 parallel reactions performed at each given concentration, which is indicated on the *x* axis. The inner line is a probit curve (dose-response rule). The outer lines are the 95% confidence interval (95% CI). Intersecting dotted lines indicate the dilution level where the estimated probability of detection is 95% (LoD95%).

To determine the LoD95% for ddPCR and qPCR, a series of two-fold dilutions of genomic DNA of the two fungi were prepared as templates at concentrations near the detection end points determined in preliminary experiments. As shown in **Figure 4**, the probit analysis of the ddPCR assay indicated a LoD95% of 6.8 copies/reaction (95% CI: 5.1–19.3) for *V. nonalfalfae* and 7.2 copies/reaction for *V. albo-atrum* (95% CI: 5.6–14.2) (**Figures 4A, B**). In contrast, the estimated LoD95% values for qPCR were slightly lower, at 5.6 copies/reaction (95% CI: 4.4–12.8) and 5.2 copies/reaction (95% CI: 4.0–15.7) for *V. nonalfalfae* and *V. albo-atrum*, respectively (**Figures 4C, D**). The results indicated that the two methods showed similar sensitivity for the pure genomic DNA of both fungi, and the resulting LoD95% values of ddPCR were slightly higher than those of qPCR for the two fungi.

### 3.4 Evaluation of the diagnostic performance of ddPCR on field samples in comparison with that of qPCR

We further investigated the applicability of ddPCR *via* a comparison with qPCR using field samples. We collected stems and roots from 60 alfalfa plants in the field, each including 10 healthy tissue controls and 50 V*. nonalfalfae*- or *V. albo-atrum*-infected samples, which had been previously confirmed.

**Figure 5:**
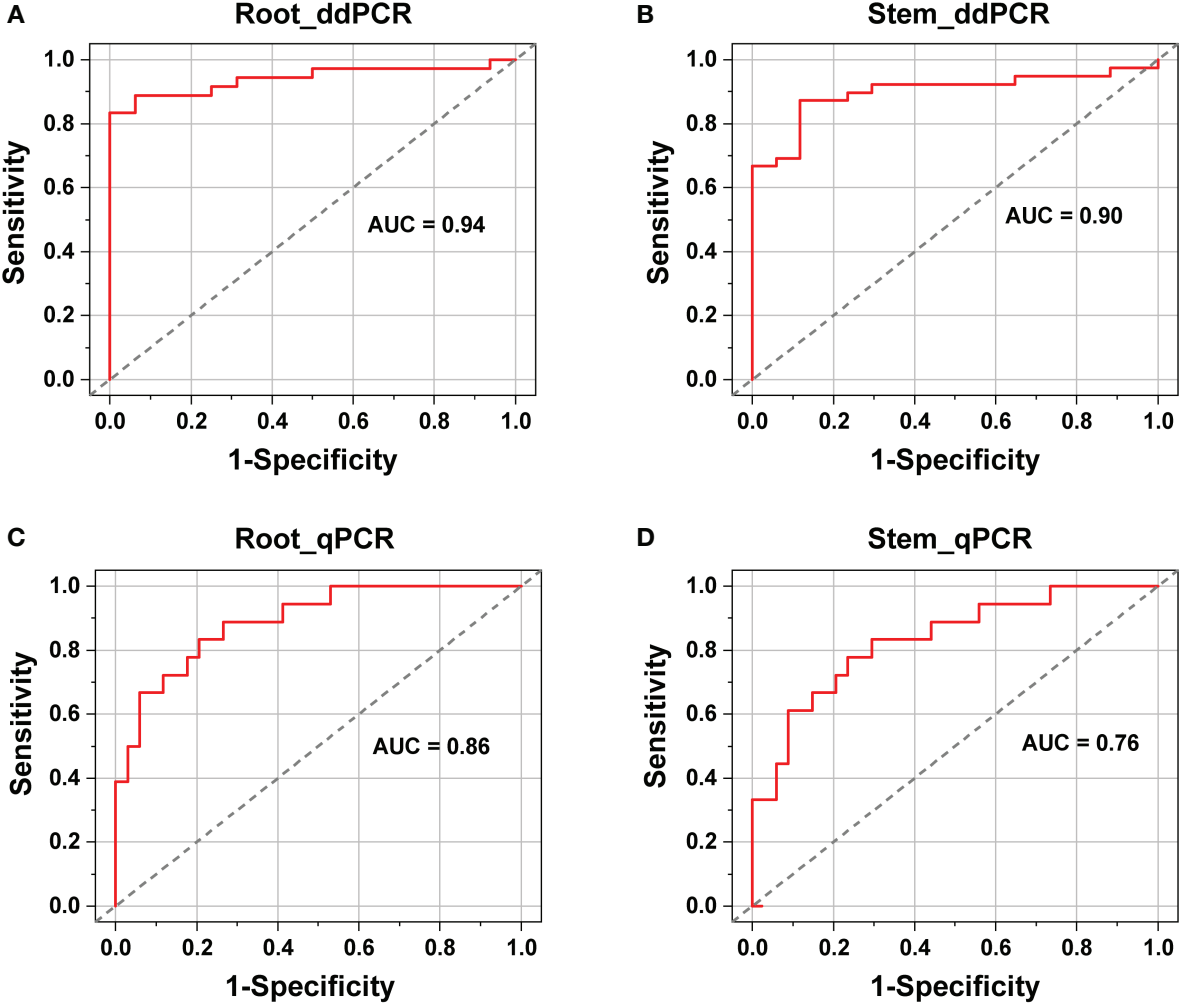
Diagnostic performance comparison between ddPCR **(A, B)** and qPCR **(C, D)** assays for the identification of healthy and infected alfalfa samples (roots and stems).

ROC analysis showed that for ddPCR, the area under curve (AUC) values were 0.94 (standard error 0.03, 95% CI: 0.88–0.99) and 0.90 (standard error 0.04, 95% CI: 0.82–0.97) for roots and stems, respectively (**Figures 5A, B**). In contrast, the AUC values for qPCR were 0.86 (standard error 0.05, 95% CI: 0.78–0.95) and 0.76 (standard error 0.06, 95% CI: 0.63–0.89) for roots and stems, respectively (**Figures 5C, D**). The AUC values of the ddPCR assays were broader than those of the qPCR assays, especially for the stems. Therefore, the ddPCR method provided a more robust diagnostic performance than that of qPCR for distinguishing the *V. nonalfalfae*- and *V. albo-atrum*-positive cases from healthy samples.

In addition, the cut-off points of the qPCR assays were determined based on the ROC curves, with values of 36 for the stem and 35 for the root samples. Among the 50 positive cases, ten stem and eight root samples were reported to be negative by qPCR. However, six out of the ten false-negative stems and four out of the eight false-negative roots tested positive by ddPCR. Furthermore, the qPCR-negative but ddPCR-positive roots and stems had an average load of 17 and 30 copies, respectively. Hence, ddPCR showed improved detection sensitivity compared to qPCR, especially regarding stem samples.

### 3.5 Evaluation of the quantification performance of ddPCR on field samples in comparison with that of qPCR

**Figure 6:**
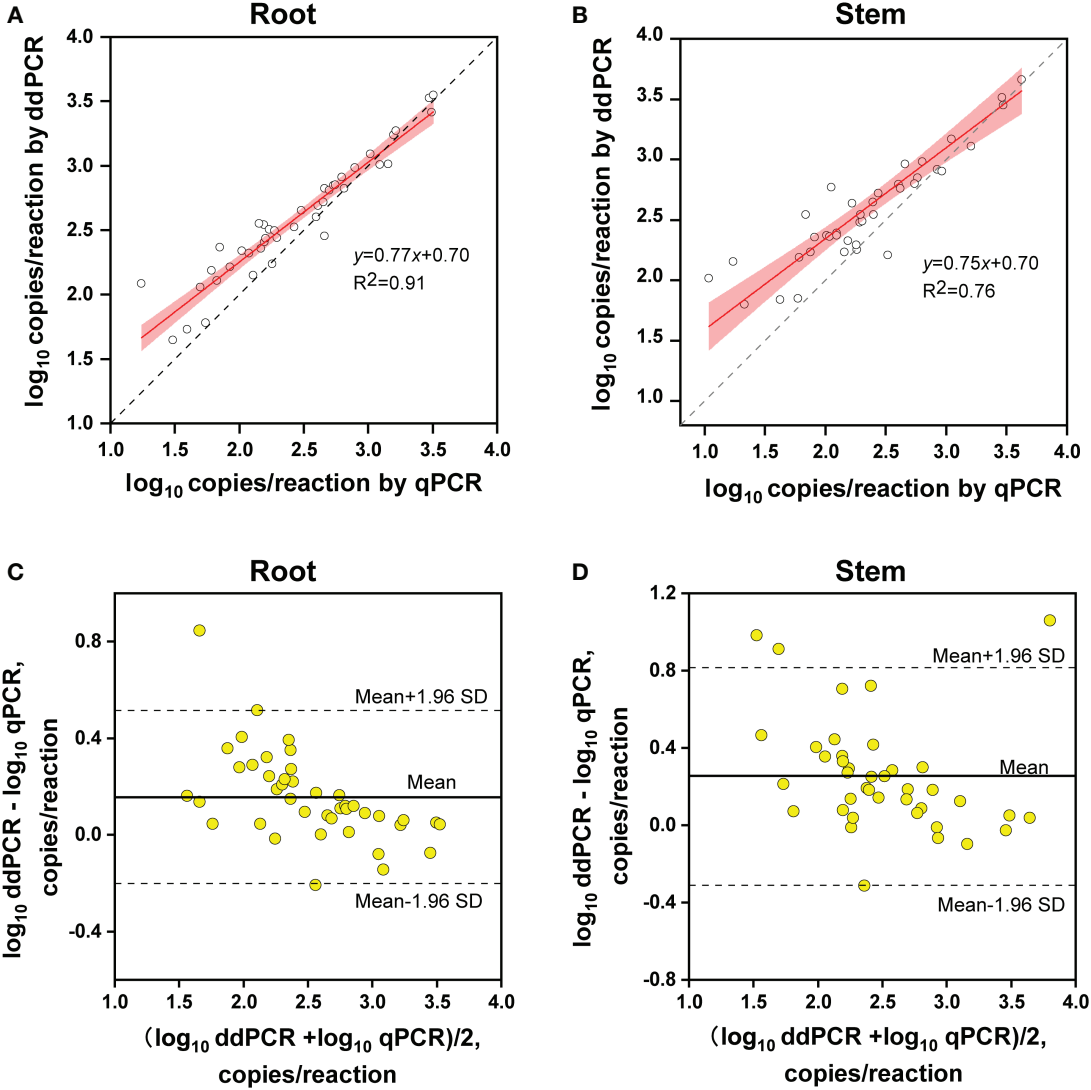
Quantitative comparison between ddPCR and qPCR. **(A, B)** Linear regression of the copy number concentration measured by ddPCR and qPCR assays in alfalfa roots **(A)** and stems **(B)**. The grey dashed lines represent the line of equality (y = x). The 95% confidence limits (red areas) are interval estimates for the regression line. **(C, D)** Bland–Altman plot analysis of the differences in log_10_ concentration for the two methods in alfalfa roots **(C)** and stems **(D)**. The solid lines in the center indicate average differences in concentration between the two methods and the dashed lines represent 95% limits of agreement (mean ± 1.96 SD) (root, n = 42; stem, n = 40).

The copy number concentrations for ddPCR and qPCR were compared using the samples that tested positive by both methods. Linear regression analysis indicated that the copy numbers determined by ddPCR correlated well with those determined by qPCR in both alfalfa tissues (roots, R^2^ = 0.91; stems, R^2^ = 0.76) (**Figures 6A, B**).

**Figure 7:**
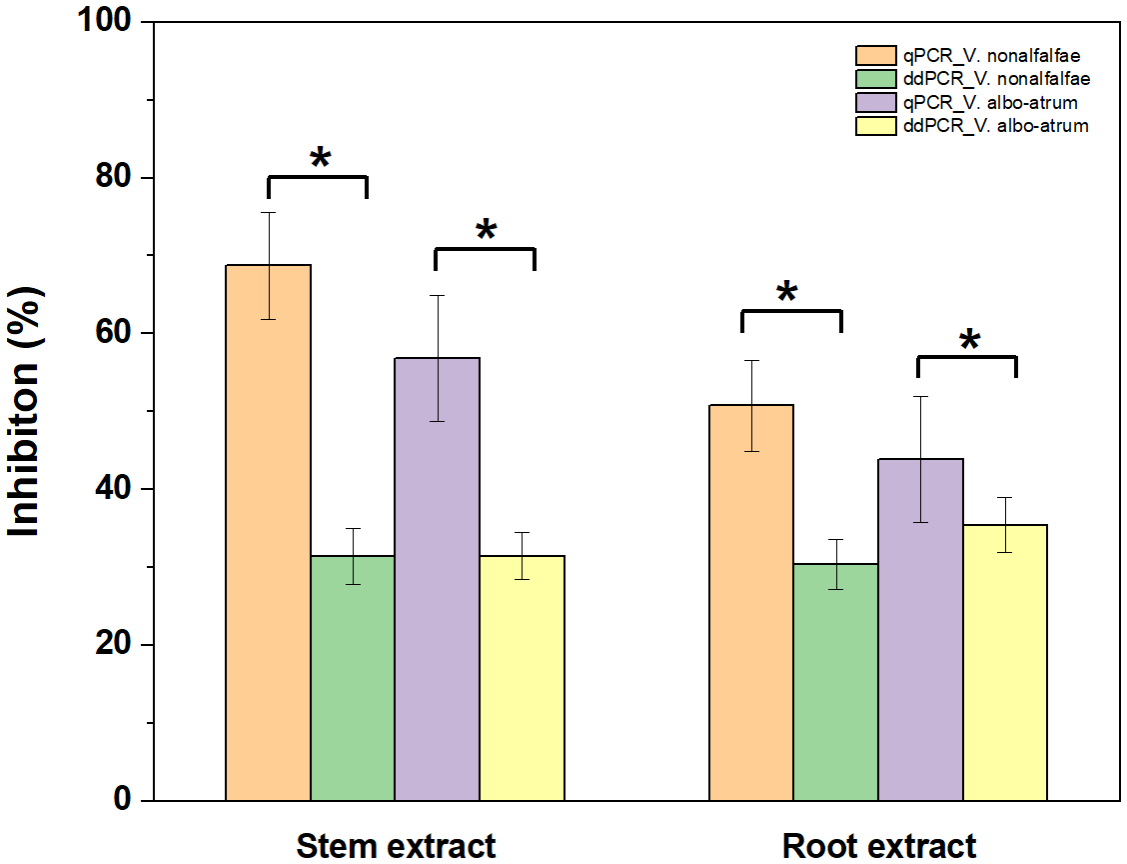
Influence of alfalfa root and stem extracts on quantification of *V. nonalfalfae* and *V. albo-atrum* by ddPCR and qPCR assays. Samples spiked with an equal amount of alfalfa root and stem extracts and genomic DNA. Error bars represent the standard error of inhibition between four replicates. Asterisks (*) indicate significantly (*p* < 0.05) different qPCR tests, compared to ddPCR.

**Figure 8:**
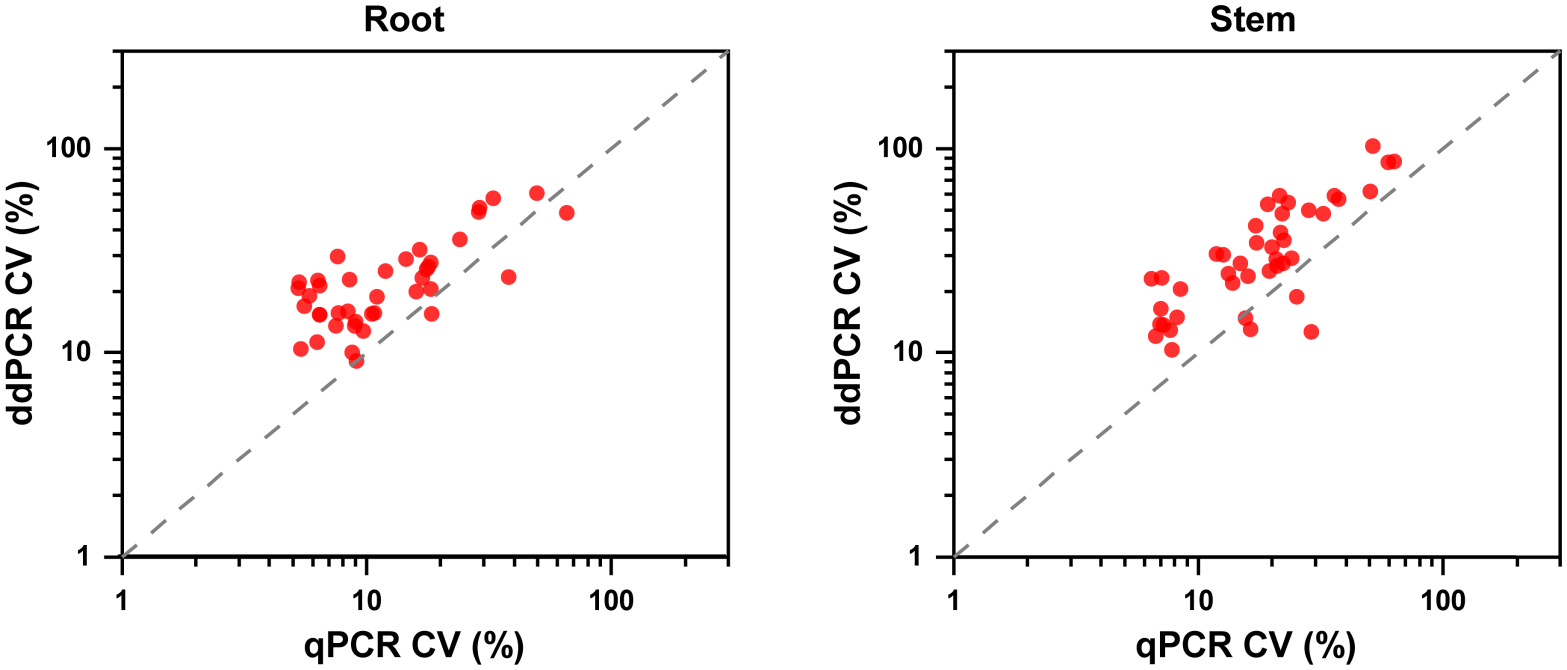
Precision experiments comparing the ddPCR and qPCR assays using CVs for the alfalfa root and stem samples (n=4).

Regarding quantitative agreement, ddPCR produced the higher copy numbers of ITS than qPCR in most tested samples. As shown in **Figures 6C, D**, Band–Altman plots showed that the bias for the agreement ranged from -0.21 to 0.51 log_10_ and -0.31 to 0.82 log_10_ for roots and stems, respectively, with most points falling inside the 95% CI. The average difference in quantification between the two methods was 0.16 log_10_ (a difference of 1.45 times on a normal scale) for roots and 0.25 log_10_ (1.78 times) for stem samples. Moreover, the intervals defined by the 95% CI for root samples were narrower than those for stem samples. To determine whether the residual matrix of field samples caused the quantification difference, we quantified the samples that spiked with equal amounts of gDNA from cultured isolates and extracts from healthy alfalfa stems and roots. The extracts inhibited the quantification of the spiked DNA by both methods, but the concentrations determined by the ddPCR were significantly higher than qPCR (**Figure 7**).In addition, absolute quantification by ddPCR revealed greater precision in *V. nonalfalfae* and *V. albo-atrum* abundance compared to those of qPCR, and the CV values were 18–72% and 28–76% lower than those of qPCR with respect to overall variation for alfalfa roots and stems, respectively (**Figure 8**).

## 4 Discussion

dPCR offers highly accurate and precise quantification by directly counting nucleic acid molecules and is commonly used as a reference measurement method to assign values for reference materials (Félix-Urquídez et al., 2016; Lee et al., 2022). In this study, we established a ddPCR assay to detect and quantify *V. nonalfalfae* and *V. albo-atrum* and compared its performance with that of the qPCR assay to investigate whether this method is more reliable for pathogen diagnosis. Overall, our data suggest that, compared to those of qPCR, ddPCR exhibits higher sensitivity, accuracy, and precision for the quantitative detection of the two fungi in field samples.

Due to sample partitioning and the end-point PCR approach, dPCR exhibits increased tolerance to suboptimal PCR reactions, such as PCR inhibitors in the samples (Dingle et al., 2013; Wang et al., 2022). In contrast, such inhibitory substances markedly influence the amplification efficiency of qPCR, introducing significant variations in its detection sensitivity and quantification accuracy (Bustin et al., 2009).

For the alfalfa field samples, ddPCR was more sensitive than qPCR for diagnosing *V. nonalfalfae* and *V. albo-atrum* at low abundances, although the two methods had similar LoD95% values when testing pure genomic DNA. We speculate that the existence of PCR-inhibitory substances in the matrix of the field samples might have been the primary cause of the reduced detection capability of qPCR; this is in agreement with previous observations (Maheshwari et al., 2017; Cao et al., 2020). Interestingly, compared to qPCR, ddPCR largely increased the AUC (from 0.76 to 0.90) for alfalfa stems, whereas for root samples, the difference between the two methods was less (0.86 versus 0.94). Alfalfa stems contain more polysaccharides, a type of amplification inhibitor, than roots do; therefore, the residual matrix inhibitors might have caused these discrepancies in detection sensitivity and diagnostic performance between pure genomic DNA and field samples.

A major advantage of dPCR over qPCR is that it enables absolute quantification without an external calibration curve, which facilitates the robustness of dPCR to variations in PCR efficiency (Sanders et al., 2011; Rački et al., 2014). In this study, the quantitative results of the positive field samples were highly concordant between methods; nevertheless, the concentrations determined by ddPCR were generally higher than those determined by qPCR, which was also found in previous studies (Maheshwari et al., 2017; Persson et al., 2018). Notably, the inhibition analysis showed that ddPCR was more tolerant to residual matrix for the quantification of targets compared with qPCR. Thus, we speculate that the interfering substances in field samples potentially markedly reduced the amplification efficiency of the qPCR, and caused the difference in PCR efficiency between the pure genomic DNA and samples, thereby resulting in the underestimation of the target concentrations (Corbisier et al., 2015). Additionally, the ddPCR assay showed appreciably decreased intra-experiment variability than those of qPCR for the most alfalfa stems and roots we examined. Hence, these strengths enable ddPCR to overcome the drawbacks of qPCR for quantitative detection in a reproducible manner.

Although this study did not further validate more target genes across multiple instruments with different sample types, our approach to ddPCR evaluation provides a valuable step toward ensuring accurate and reliable analytical and field performance for the quantitative monitoring of plant pathogens. Currently, the application of dPCR is limited, largely owing to its complicated operational workflow and high cost. Considering that these issues will likely improve with ongoing technological development, we consider that dPCR methods will play an increasingly important role in sensitive and accurate quantitative detection in various fields.

## 5 Conclusions

In conclusion, this study aimed to develop and validate a sensitive and accurate ddPCR assay to quantitatively detect *V. nonalfalfae* and *V. albo-atrum*. Our results demonstrate that the dPCR method is sensitive and robust for detecting field samples with low-titer pathogens and residual matrix inhibitors. Furthermore, it can provide an accurate and reproducible alternative to qPCR for diagnostic applications. Finally, dPCR is of higher metrological quality, which facilitates measurement standardization and can be used for inter-laboratory comparisons.

## Data availability statement

The datasets presented in this study can be found in online repositories. The names of the repository/repositories and accession number(s) can be found in the article/**Supplementary Material**.

## Author contributions

Conceptualization, XS and YG. Methodology, DW and EL. Software, XJ and CN. Data curation, EL, HL, and CN. Writing-original draft preparation, review and editing, DW, YG and XS. Funding acquisition, DW, HL and XS. All authors have read and agreed to the published version of the manuscript.

## References

[B1] Alikian M. Whale A. S. Akiki S. Piechocki K. Torrado C. Myint T.. (2017). RT-qPCR and RT-digital PCR: A comparison of different platforms for the evaluation of residual disease in chronic myeloid leukemia. Clin. Chem. 63, 525–531. doi: 10.1373/clinchem.2016.262824 27979961

[B2] Baker M. (2012). Digital PCR hits its stride. Nat. Methods 9, 541–544. doi: 10.1038/nmeth.2027

[B3] Bland J. M. Altman D. G. (1986). Statistical methods for assessing agreement between two methods of clinical measurement. Lancet 1, 307–310. doi: 10.1016/S0140-6736(86)90837-8 2868172

[B4] Bustin S. A. Benes V. Garson J. A. Hellemans J. Huggett J. Kubista M.. (2009). The MIQE guidelines: Minimum information for publication of quantitative real-time PCR experiments. Clin. Chem. 55, 611–622. doi: 10.1373/clinchem.2008.112797 19246619

[B5] Cao W. Li Y. Chen X. Chang Y. Li L. Shi L.. (2020). Species identification and quantification of silver pomfret using the droplet digital PCR assay. Food Chem. 302, 125331–125337. doi: 10.1016/j.foodchem.2019.125331 31404867

[B6] Corbisier P. Pinheiro L. Mazoua S. Kortekaas A. M. Chung P. Y. J. Gerganova T.. (2015). DNA copy number concentration measured by digital and droplet digital quantitative PCR using certified reference materials. Anal. Bioanal. Chem. 407, 1831–1840. doi: 10.1007/s00216-015-8458-z 25600685 PMC4336415

[B7] Corman V. M. Landt O. Kaiser M. Molenkamp R. Meijer A. Chu D. K.. (2020). Detection of 2019 novel coronavirus, (2019-nCoV) by real-time RT-PCR. Euro. Surveill. 25, 2000045–52. doi: 10.2807/1560-7917.es.2020.25.3.2000045 31992387 PMC6988269

[B8] Dingle T. C. Sedlak R. H. Cook L. Jerome K. R. (2013). Tolerance of droplet-digital PCR vs real-time quantitative PCR to inhibitory substances. Clin. Chem. 59, 1670–1672. doi: 10.1373/clinchem.2013.211045 24003063 PMC4247175

[B9] Félix-Urquídez D. Pérez-Urquiza M. Valdez Torres J. B. León-Félix J. García-Estrada R. Acatzi-Silva A. (2016). Development, optimization, and evaluation of a duplex droplet digital pcr assay to quantify the T-nos/hmg copy number ratio in genetically modified maize. Anal. Chem. 88, 812–819. doi: 10.1021/acs.analchem.5b03238 26605751 PMC4718530

[B10] Flajsman M. Radisek S. Javornik B. (2017). Pathogenicity assay of *Verticillium nonalfalfae* on hop plants. Bio Protoc. 7, e2171–e2180. doi: 10.21769/BioProtoc.2171 PMC8376619 34458482

[B11] Floerl S. Druebert C. Majcherczyk A. Karlovsky P. Kües U. Polle A. (2008). Defence reactions in the apoplastic proteome of oilseed rape (*Brassica napus* var. napus) attenuate *Verticillium longisporum* growth but not disease symptoms. BMC Plant Biol. 8, 129–129. doi: 10.1186/1471-2229-8-129 19094241 PMC2644697

[B12] Forootan A. Sjoback R. Bjorkman J. Sjogreen B. Linz L. Kubista M. (2017). Methods to determine limit of detection and limit of quantification in quantitative real-time PCR (qPCR). Biomol. Detect Quantif. 12, 1–6. doi: 10.1016/j.bdq.2017.04.001 28702366 PMC5496743

[B13] Fradin E. F. Thomma B. P. (2006). Physiology and molecular aspects of Verticillium wilt diseases caused by *V. dahliae and V. albo-atrum* . Mol. Plant Pathol. 7, 71–86. doi: 10.1111/j.1364-3703.2006.00323.x 20507429

[B14] General Administration of Quality Supervision, Inspection and Quarantine of the People’s Republic of China (2014). Detetion and identification of Verticillium albo-atrum Reinke & Berthold. SN/T 1145-2014.

[B15] General Administration of Customs of the People's Republic of China (2019). Detetion and identification of Verticillium dahliae Kleb. SN/T 5138–2019.

[B16] Griffiths D. A. (1971). The development of lignitubers in roots after infection by *Verticillium dahliae* kleb. Can. J. Microbiol. 17, 441–444. doi: 10.1139/m71-074 4101737

[B17] Hindson C. M. Chevillet J. R. Briggs H. A. Gallichotte E. N. Ruf I. K. Hindson B. J.. (2013). Absolute quantification by droplet digital PCR versus analog real-time PCR. Nat. Methods 10, 1003–1005. doi: 10.1038/nmeth.2633 23995387 PMC4118677

[B18] Huggett J. F. Cowen S. Foy C. A. (2015). Considerations for digital PCR as an accurate molecular diagnostic tool. Clin. Chem. 61, 79–88. doi: 10.1373/clinchem.2014.221366 25338683

[B19] Isaac I. (1946). Verticillium wilt of sainfoin. Ann. Appl. Biol. 33, 28–34. doi: 10.1111/j.1744-7348.1946.tb06269.x 20997932

[B20] Klosterman S. J. Subbarao K. V. Kang S. Veronese P. Gold S. E. Thomma B. P.. (2011). Comparative genomics yields insights into niche adaptation of plant vascular wilt pathogens. PloS Pathog. 7, e1002137–1002155. doi: 10.1371/journal.ppat.1002137 21829347 PMC3145793

[B21] Kralik P. Ricchi M. (2017). A basic guide to real time PCR in microbial diagnostics: definitions, parameters, and everything. Front. Microbiol. 8. doi: 10.3389/fmicb.2017.00108 PMC5288344 28210243

[B22] Larsen R. C. Vandemark G. J. Hughes T. J. Grau C. R. (2007). Development of a real-time polymerase chain reaction assay for quantifying *Verticillium albo-atrum* DNA in resistant and susceptible alfalfa. Phytopathology 97, 1519–1525. doi: 10.1094/PHYTO-97-11-1519 18943523

[B23] Lee S. S. Kim S. Yoo H. M. Lee D. H. Bae Y. K. (2022). Development of SARS-CoV-2 packaged RNA reference material for nucleic acid testing. Anal. Bioanal. Chem. 414, 1773–1785. doi: 10.1007/s00216-021-03846-y 34958396 PMC8711077

[B24] Maheshwari Y. Selvaraj V. Hajeri S. Yokomi R. (2017). Application of droplet digital PCR for quantitative detection of spiroplasma citri in comparison with real time PCR. PloS One 12, e0184751–0184766. doi: 10.1371/journal.pone.0184751 28910375 PMC5599046

[B25] Maurer K. A. Radišek S. Berg G. Seefelder S. (2013). Real-time PCR assay to detect *Verticillium albo-atrum and V. dahliae* in hops: Development and comparison with a standard PCR method. J. Plant Dis. Protect 120, 105–114. doi: 10.1007/BF03356461

[B26] Pasche J. S. Mallik I. Anderson N. R. Gudmestad N. C. (2013). Development and validation of a real-time PCR assay for the quantification of *Verticillium dahliae* in potato. Plant Dis. 97, 608–618. doi: 10.1094/PDIS-06-12-0554-RE 30722203

[B27] Pavšič J. Žel J. Milavec M. (2016). Assessment of the real-time PCR and different digital PCR platforms for DNA quantification. Anal. Bioanal. Chem. 408, 107–121. doi: 10.1007/s00216-015-9107-2 26521179 PMC4706846

[B28] Pegg G. F. Brady B. L. (2002). Verticillium wilts Vol. 151 (CABI Publishing), 109–110. doi: 10.1079/9780851995298.0000

[B29] Persson S. Eriksson R. Lowther J. Ellstrom P. Simonsson M. (2018). Comparison between RT droplet digital PCR and RT real-time PCR for quantification of noroviruses in oysters. Int. J. Food Microbiol. 284, 73–83. doi: 10.1016/j.ijfoodmicro.2018.06.022 30005929

[B30] Pinheiro L. B. Coleman V. A. Hindson C. M. Herrmann J. Hindson B. J. Bhat S.. (2012). Evaluation of a droplet digital polymerase chain reaction format for DNA copy number quantification. Anal. Chem. 84, 1003–1011. doi: 10.1021/ac202578x 22122760 PMC3260738

[B31] Rački N. Morisset D. Gutierrez-Aguirre I. Ravnikar M. (2014). One-step RT-droplet digital PCR: a breakthrough in the quantification of waterborne RNA viruses. Anal. Bioanal. Chem. 406, 661–667. doi: 10.1007/s00216-013-7476-y 24276251 PMC3892107

[B32] Salipante S. J. Jerome K. R. (2020). Digital PCR-an emerging technology with broad applications in microbiology. Clin. Chem. 66, 117–123. doi: 10.1373/clinchem.2019.304048 31704712

[B33] Sanders R. Huggett J. F. Bushell C. A. Cowen S. Scott D. J. Foy C. A. (2011). Evaluation of digital PCR for absolute DNA quantification. Anal. Chem. 83, 6474–6484. doi: 10.1021/ac103230c 21446772

[B34] Skadow K. (1966). Weeds as host-plants for *Verticillium albo-atrum* rke. et berth. including *Verticillium dahliae* kleb. Zentralbl. Bakteriol. Parasitenkd Infektionskr Hyg. 120, 49–59. 5953031

[B35] Su X. Lu G. Rehman L. Li X. Sun L. Guo H.. (2018). *mcherry*-labeled *Verticillium dahliae* could be utilized to investigate its pathogenicity process in *Nicotiana benthamiana* . Genes 9, 508–520. doi: 10.3390/genes9100508 30340423 PMC6210675

[B36] Svec D. Tichopad A. Novosadova V. Pfaffl M. W. Kubista M. (2015). How good is a PCR efficiency estimate: Recommendations for precise and robust qPCR efficiency assessments. Biomol. Detect Quantif. 3, 9–16. doi: 10.1016/j.bdq.2015.01.005 27077029 PMC4822216

[B37] Wang D. CHen J. Y. Song J. Li J. Klosterman S. J. Li R.. (2021). Cytotoxic function of xylanase VdXyn4 in the plant vascular wilt pathogen *Verticillium dahliae* . Plant Physiol. 187, 1–21. doi: 10.1093/plphys/kiab274 34618145 PMC8418393

[B38] Wang D. Jiao X. Jia H. Cheng S. Jin X. Wang Y.. (2022). Detection and quantification of *Verticillium dahliae and V. longisporum* by droplet digital PCR versus quantitative real-time PCR. Front. Cell Infect. Microbiol. 12. doi: 10.3389/fcimb.2022.995705 PMC9441566 36072220

[B39] Whale A. S. Huggett J. F. Cowen S. Speirs V. Shaw J. Ellison S.. (2012). Comparison of microfluidic digital PCR and conventional quantitative PCR for measuring copy number variation. Nucleic Acids Res. 40, e82–e90. doi: 10.1093/nar/gks203 22373922 PMC3367212

[B40] Zhao P. Zhao Y. L. Jin Y. Zhang T. Guo H. S. (2014). Colonization process of *Arabidopsis thaliana* roots by a green fluorescent protein-tagged isolate of *Verticillium dahliae* . Protein Cell 5, 94–98. doi: 10.1007/s13238-013-0009 24481631 PMC3956967

