# Supplementary material for: A droplet digital PCR assay for detection and quantification of Verticillium nonalfalfae and V. albo-atrum
Source: Front Cell Infect Microbiol. 2023 Jan 11;12:1110684. doi: 10.3389/fcimb.2022.1110684 (PMC9874294; doi:10.3389/fcimb.2022.1110684)
Supplement: Supplementary file 1 [file DataSheet_1.docx]

Supplementary Material

A droplet digital PCR assay for detection and quantification of *Verticillium nonalfalfae* and *Verticillium albo-atrum*

Di Wang ^1,†^, Enliang Liu ^2,†^, Haiyang Liu ^3^, Xi Jin ^4^, Chunyan Niu ^1^, Yunhua Gao^1,^* and Xiaofeng Su ^5,^*

*** Correspondence:** Xiaofeng Su: suxiaofeng@caas.cn (X.S.); Yunhua Gao:gaoyh@nim.ac.cn (Y.G.)

**Table S1.** The Ct values obtained by different primer/probe sets

| **Primer/probe**  **DNA** | **Amount (ng)** | **Va1** | **Va2** | **Va3** | **Va4** |
| --- | --- | --- | --- | --- | --- |
| *V. nonalfalfae* | 0.2 | 28.44 | Un | Un | 15.06 |
| *V. albo-atrum* | 0.3 | 25.42 | Un | Un | 13.96 |

Table S2. Specificity test for Va4-ddPCR assay

| **Name** | **Ct value** |
| --- | --- |
| *Verticillium dahliae* | Un. |
| *Verticillium longisporum* | Un. |
| *Verticillium nigrescens* | Un. |
| *Magnaporthe oryzae* | Un. |
| *Bipolaris maydis* | Un. |
| *Exserohilum turcicum* | Un. |
| *Fusarium oxysporum f. sp. conglutinans* | Un. |
| *Rhizoctonia cerealis* | Un. |
| *Meloidogyne incognita* | Un. |
| *Fusarium pseudograminearum* | Un. |
| *Ustilaginoidea virens* | Un. |
| *Acidovorax citrulli* | Un. |
| *Xanthomonas oryzae pv. oryzae* | Un. |
| *Pseudomonas syringae* | Un. |
| *Ralstonia solanacearum* | Un. |
| *Xanthomonas campestris pv.campestris* | Un. |


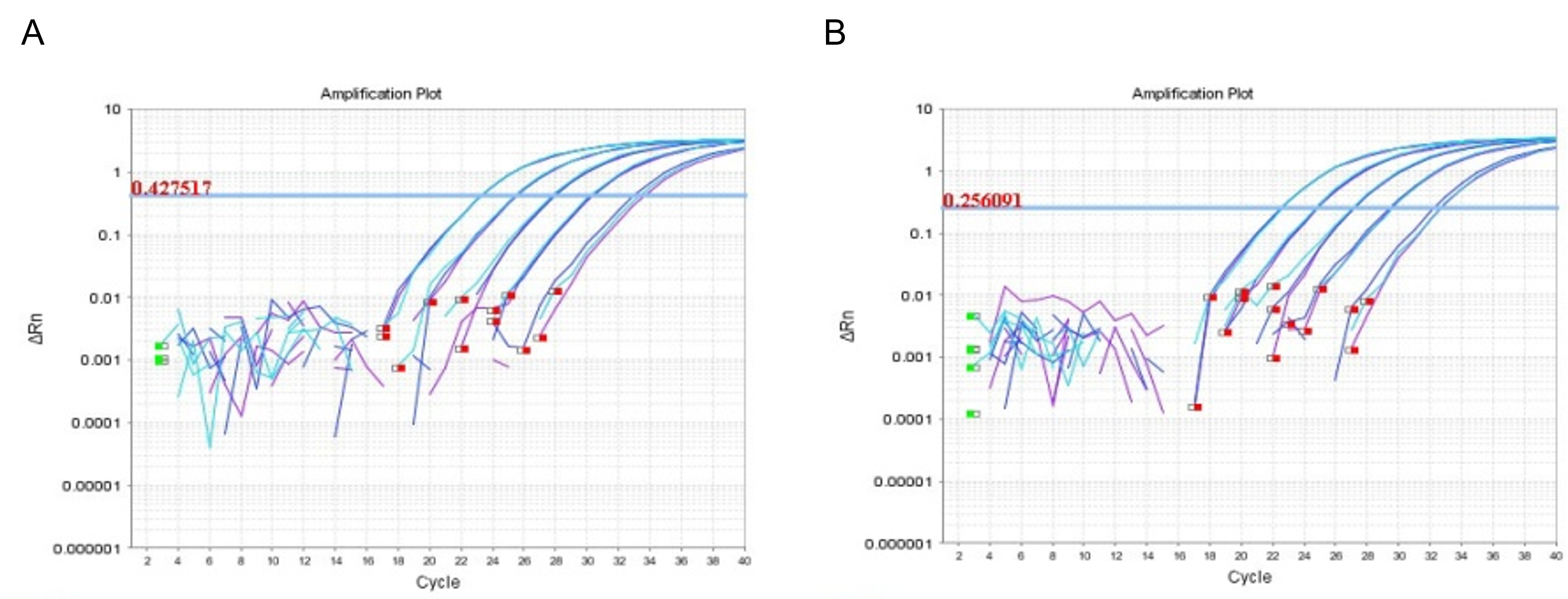


**Figure S1**. Amplified curves of qPCR assays using Va4 primer/probe set to detect *V. nonalfalfae* (A) and *V. albo-atrum* (B).
